# Supplementary material for: A domain in human EXOG converts apoptotic endonuclease to DNA-repair exonuclease
Source: Nat Commun. 2017 May 3;8:14959. doi: 10.1038/ncomms14959 (PMC5418593; doi:10.1038/ncomms14959)
Supplement: Supplementary Information — Supplementary Figures and Supplementary Tables [file ncomms14959-s1.pdf]

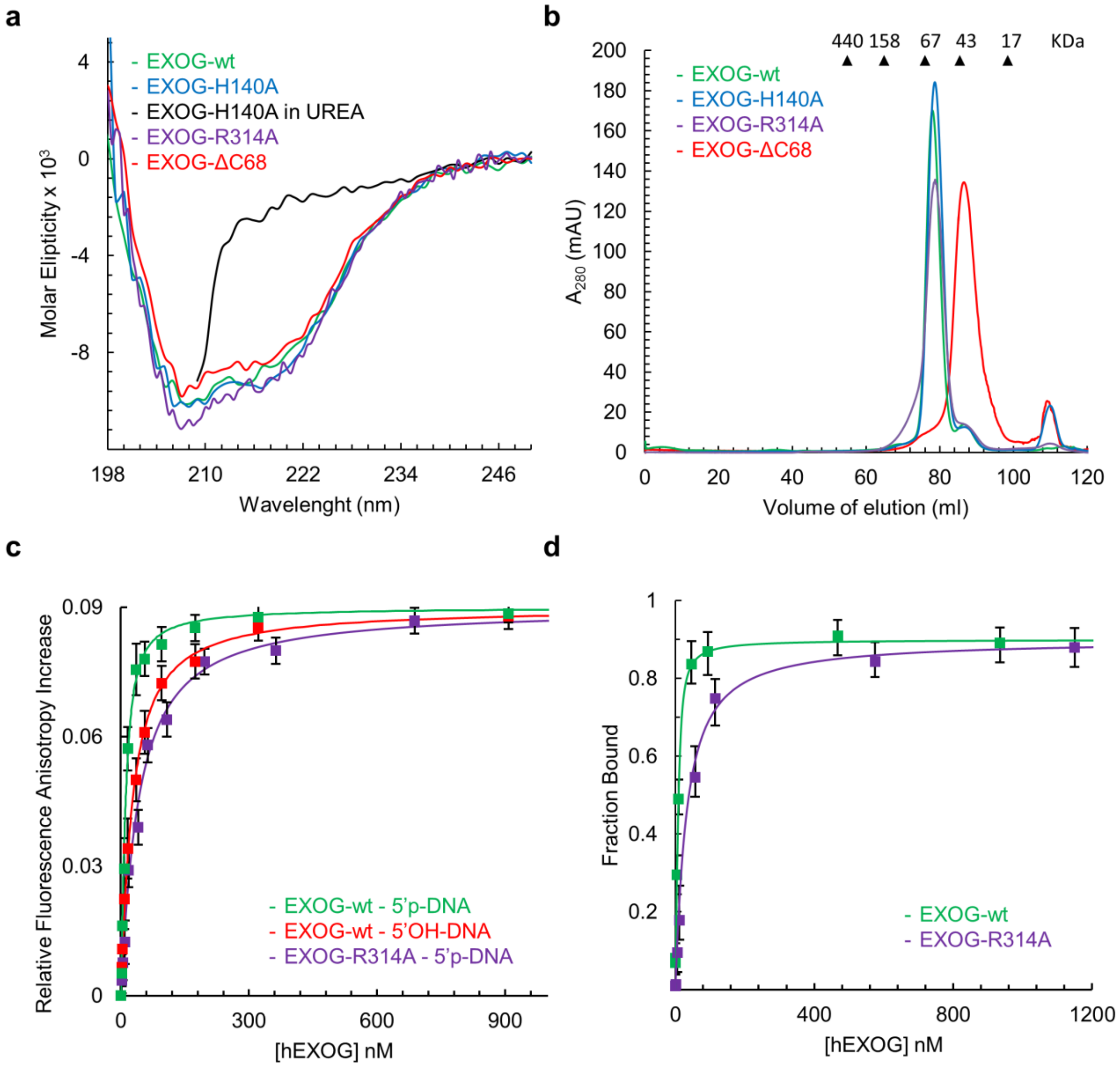

Supplementary Figure 1. Protein folding, purification and DNA binding.
(a) CD spectra for folded hEXOG variants and unfolded hEXOG-H140A in 8M urea.
(b) Overlay of gel filtration elution profiles with molecular weight standards for hEXOG-wt (green), hEXOG-H140A (blue), hEXOG-ΔC68 (red) and hEXOG-R314A (magenta) on a Superdex 200 HiLoad 16/60 column.
(c) Binding to 10 bp DNA (10 nM) containing 5′P or 5′OH measured by fluorescence anisotropy. Apparent K_d values are determined by fitting using Mathematica. Apparent Kd for wild-type - 5′P-DNA and -5′OH-DNA are 6.25 ± 2.0 nM and 22.2 ± 5.1 nM, respectively. Apparent Kd for R314A mutant - 5′P-DNA is 35.7 ± 7.2 nM.
(d) Binding to 10 bp DNA (10 nM) measured by EMSA. Apparent K_d values for wild-type and R314A mutant, determined by fitting using Mathematica, are to be 3.4 ± 1.2 nM and 26.5 ± 6.6 nM for wild-type and R314A hEXOG, respectively. Data represent the mean ± s.e.m. of three independent experiments.

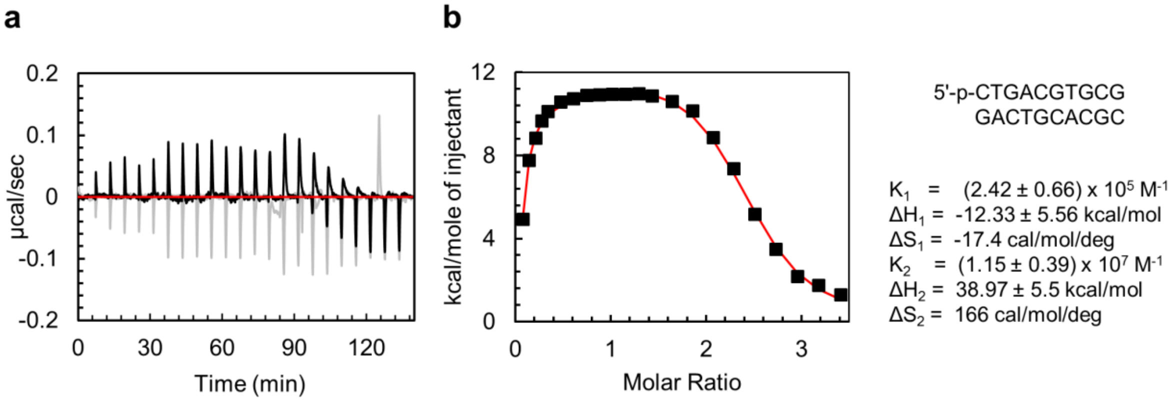

Supplementary Figure 2. Isothermal titration calorimetry of hEXOG-DNA interactions.
(a) Representative thermograms for heat release upon titration of 5′P-dsDNA into hEXOG-H140A. Heat release upon binding (black) was corrected by reference titration to account for the heat of dilution (gray).
(b) Binding isotherm created by plotting the integrated heat peaks against the molar ratio of DNA to hEXOG-H140A dimer. Thermodynamic binding parameters derived from fitting isotherms to a model with two sequential binding sites.

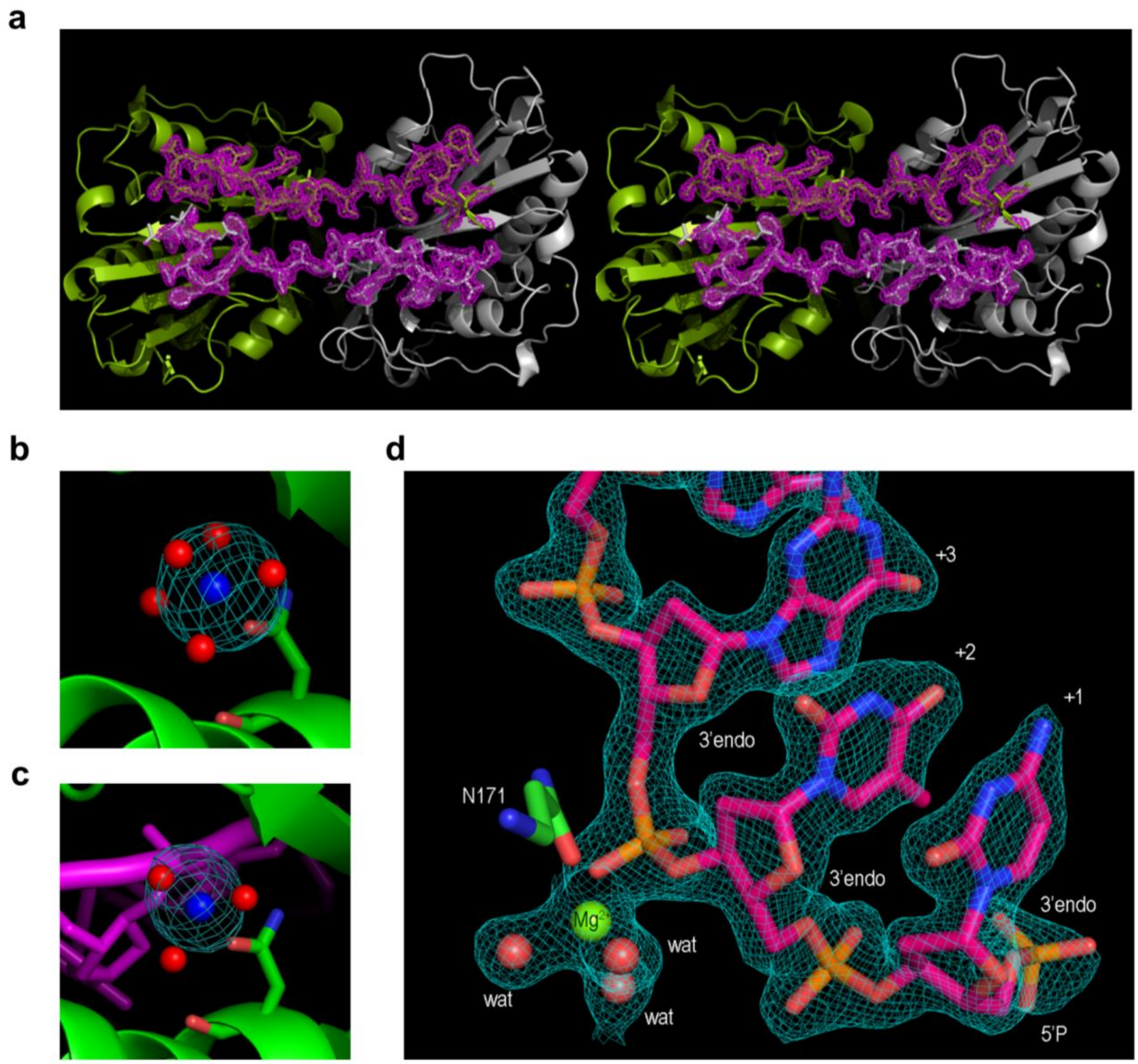

Supplementary Figure 3. Electron density maps.

(a) Stereo view of domain-swap region with electron density map (sigma weighted m2Fo-Fc map) contoured at 1.2 σ.

(b) Anomalous difference Fourier map (contoured at 5 σ) of hEXOG-Mn^{2+} complex displayed around the metal ion site (blue sphere).

(c) Anomalous difference Fourier map (contoured at 5 σ) of hEXOG-Mn^{2+}-DNA complex displayed around the metal ion site (blue sphere).

(d) Electron density 2Fo-Fc map (contoured at 1.2 σ) around substrate in the hEXOG-DNA complex. Nucleotides +1 to +3 adopt a 3'-endo sugar pucker configuration. Mg^{2+} ion forms an octahedral coordination with three water molecules, N^{171}, one bridging and one non-bridging oxygen.

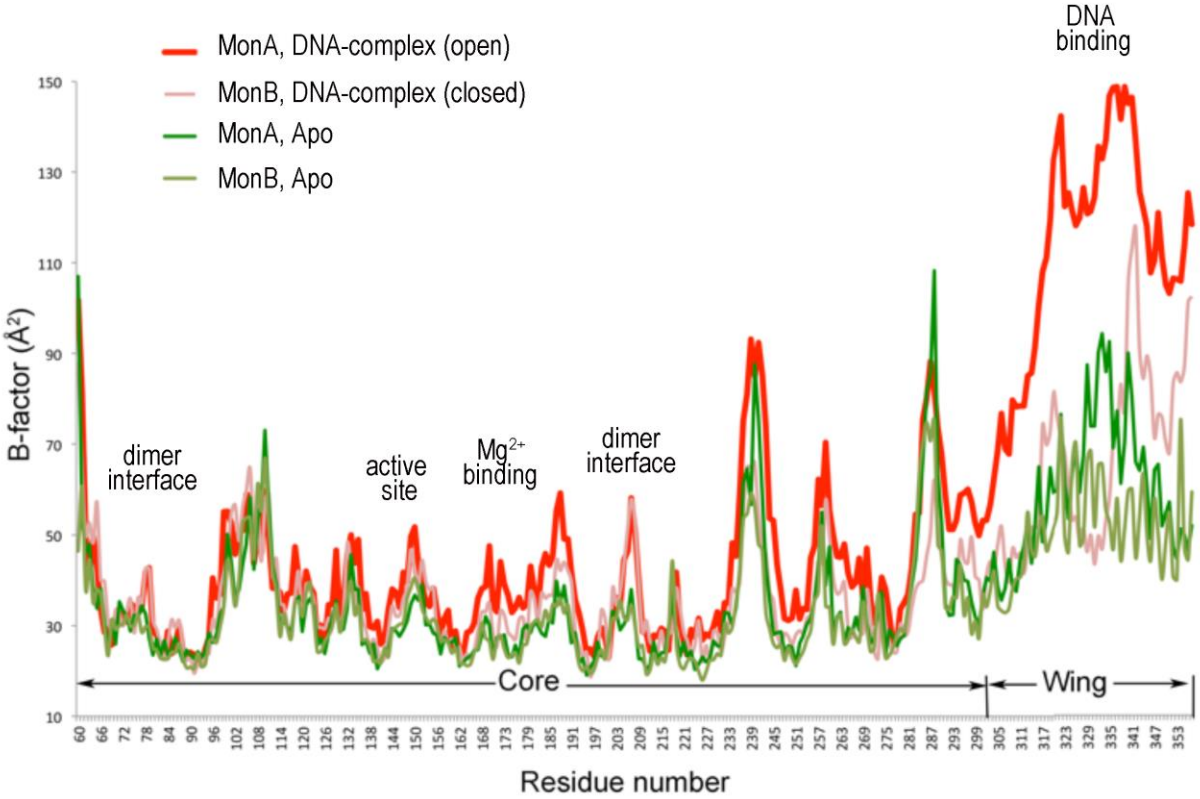

Supplementary Figure 4. Thermal motion of the Wing domain induced by DNA-binding. The B-factors for the Cα backbones of each monomer in the apo enzyme (dark and light green) are comparable but deviate in the DNA-bound form. The closed Wing domain (pink) has an averaged B-factor value similar to the apo enzyme, but the open Wing domain (red) has a significant increase.

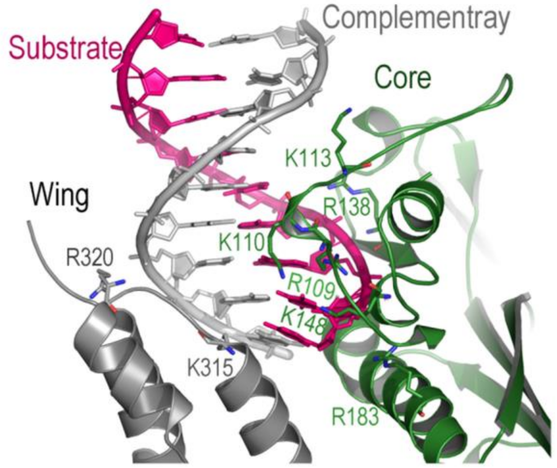

Supplementary Figure 5. Differential hEXOG-DNA interactions.
The Core domain primarily contacts the substrate strand (magenta), and the Wing domain contacts the complementary strand (gray).

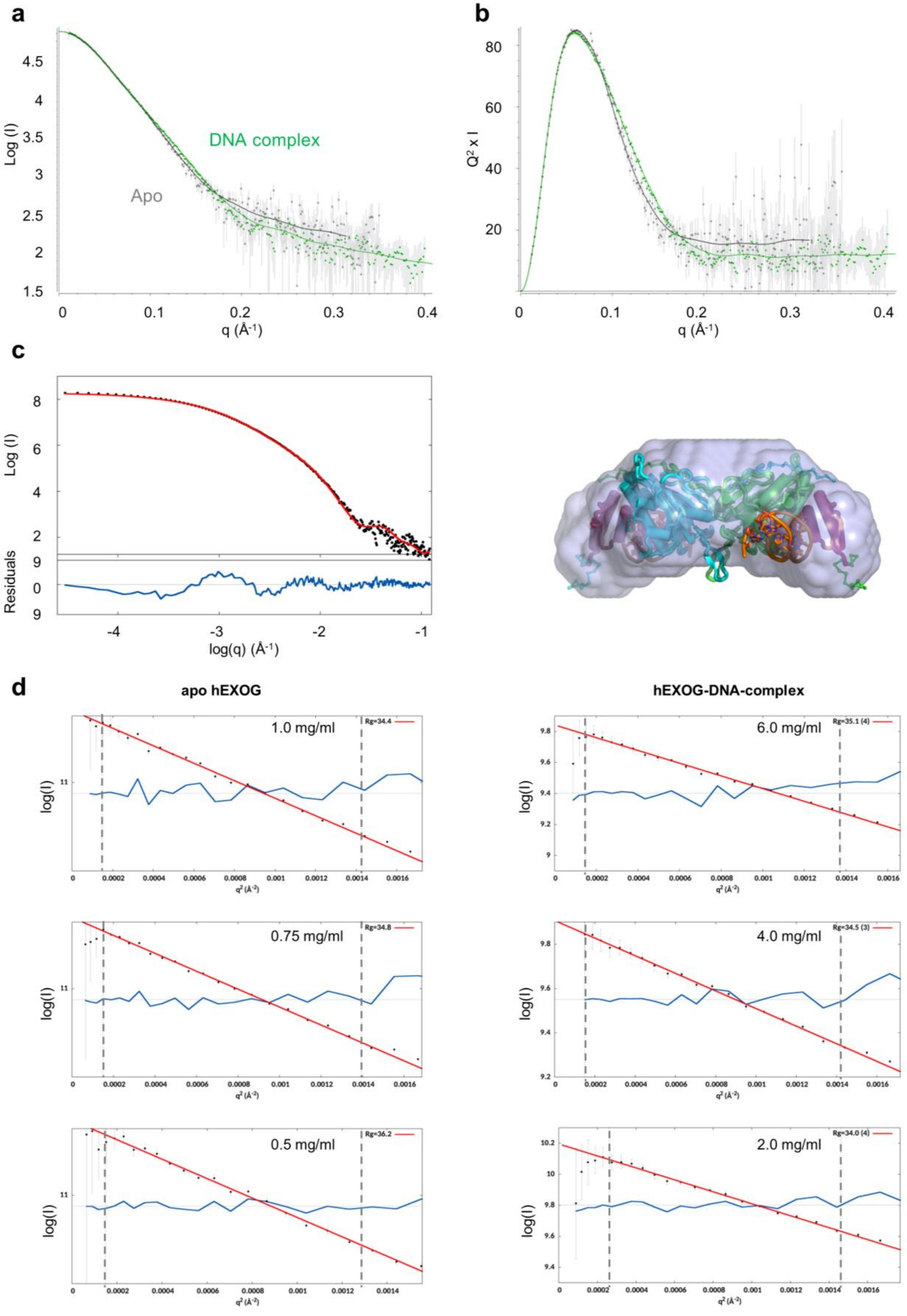

a
DNA complex
Apo
Log (I)
q (Å-1)
b
Q2 x I
q (Å-1)
c
Log (I)
Residuals
log(q) (Å-1)
d
apo hEXOG
hEXOG-DNA-complex
1.0 mg/ml
Rg=34.4
0.75 mg/ml
Rg=34.8
0.5 mg/ml
Rg=36.2
6.0 mg/ml
Rg=35.1 (4)
4.0 mg/ml
Rg=34.5 (3)
2.0 mg/ml
Rg=34.0 (4)
log(I)
q2 (Å-2)

Supplementary Figure 6. Small Angle X-ray Scattering (SAXS).

(a) Apo and hEXOG-DNA complex Scattering curves. The curves are calculated from data shown in (d). The curve for apo hEXOG (gray) is similar to that of hEXOG-DNA complex (green) , except for significant differences in their mid-q regions. The R_G and D_{MAX} values for the hEXOG apo form and DNA complex are 35.0 Å and 33.8 Å, 122 Å and 120 Å, respectively.

(b) Kratky plot of the same data. The overall cross-chi square is 2.2.

(c) Log-log plot of merged SAXS data for hEXOG-DNA complex with CORAL 2 domain model (χ_2=1.3). The *ab initio* SAXS model (surface) is in good agreement with the CORAL 2 domain model (cartoon representation).

(d) Guinier plots for apo and DNA bound hEXOG at indicated concentrations. The red line is the Guinier fit and the blue line normalized residuals (residual/error). The q-values used for Guinier fitting range from 0.0122 to 1.3/Rg ($Å^{-1}$) for all concentrations with exception of the one at 2.0mg/ml that was from 0.0162 to 1.3/Rg ($Å^{-1}$), indicated between two vertical dashed lines, as determined by PRIMUS.

Supplementary Table 1. Sequences of oligonucleotides

	Name	Sequence
ITC / Fluorescence Crystallization Activity assays	10-mer #55	5′-p-CTG ACG TGC G-3′
	10-mer #62	5′-OH-CTG ACG TGC G-3′
	10-mer #43	5′-CGC ACG TCA G-3′
	20-mer #94	5′-p-CGC ACG TCA GGC AGG CTC GT-F-3′
	20-mer #54	5′-p-CGC ACG TCA GGC AGG CTC GT-3′
	20-mer #59	5′-ACG AGC CTG CCT GAC GTG CG-3′
	24-mer #7	5′-CGA AAA CGA GGG CCA GTG CCA TAC -3′
	45-mer #107	5′-ACG AGC CTG CCT GAC GTG CGA GTA TGG CAC TGG CCC TCG TTT TCG-3′

10 bp 5′P-dsDNA consists of 10-mer #55 and 10-mer #43

10 bp 5′OH-dsDNA consists of 10-mer #62 and 10-mer #43

20 bp $5'^{32}$P-dsDNA used in activity assays consists 20-mer #54 and 20-mer #59

20 bp 5′P-dsDNA-3′F consists of 20-mer #94 and 20-mer #59

The gapped-DNA consists of 45-mer #107, 24-mer #7 and 20-mer #59

Supplementary Table 2. Quantification of the burst product

**[hEXOG $_{monomer}$], nM**	**22**	**36**	**50**	**66**
**[burst product] (A0), nM**	**10.7**	**14.4**	**22.8**	**38.9**
**[A0]/[hEXOG$_{monomer}$]**	**0.49**	**0.40**	**0.46**	**0.59**

Supplementary Table 3. Classification of each dinucleotide step in a right-handed nucleic acid structure by 3DNA analysis

**Step**	**Xp**	**Yp**	Zp	**XpH**	**YpH**	**ZpH**	**Form**
1 CT/AG	-2.68	8.57	1.50	-5.27	8.23	2.90	A
2 TG/CA	-2.58	9.01	1.21	-6.74	8.31	3.65	A
3 GA/TC	-2.28	8.72	0.99	-3.26	8.76	0.56	A
4 AC/GT	-3.01	9.18	0.32	-4.51	9.17	0.61	B
5 CG/CG	-2.42	8.94	0.44	-1.91	8.95	-0.07	B
6 GT/AC	-2.76	9.22	-0.05	-3.60	9.21	0.44	B
7 TG/CA	-2.11	9.02	-0.09	-1.15	8.97	-0.93	B
8 GC/GC	-2.40	9.12	0.42	-3.17	9.03	1.02	B
9 CG/CG	-4.11	8.64	0.51	-4.69	8.55	-1.07	B
